# Acute Laryngitis

**Published:** 1852-11

**Authors:** 


*Acute Laryngitis.*—Inclosed you have an exceedingly graphic account of a very interesting case, which you are at liberty to present to the profession should you deem it worthy of publication.

The extreme asphyxia, verging on death, presented to the surgeon, the primary operation, its absolute success and efficiency, and the *length of time* which the tube has been worn with apparent impunity to the surrounding parts, are truly extraordinary features in the narration.

I would simply remark that the account is drawn up entirely by my friend and patient, Mr. Hamlin, of this city, late a highly worthy and respectable merchant of Philadelphia, and that his statements are implicitly reliable.

In conclusion, permit me to say, that although his general health, now fair, continues to improve, and physical system to develope, the local stricture yet presents a case of peculiar interest both to the patient and to science. We trust, however, that continued perseverance and care may yet overcome the remaining obstacle to complete success, and that he may be again restored to health and his accustomed occupation and usefulness.

With great respect, believe me truly yours, &c.,

PROVIDENCE, R. I., July 29, 1852. J. M.

P. S.—Should be happy to receive hints from the profession adapted practically to the case. M.

DR. JOSEPH MAURAN, Providence:

*Dear Sir*,—Supposing that a history of my case might prove of interest to you, I take this occasion to give a statement from the period of the first attack until the present time.

I have been subject to an occasional sore throat for the past eight or ten years, which heretofore had yielded to the prescribed remedies.

In the month of January, of this year, a violent cold left me with hoarseness, which gave but little trouble at first, and was of so light a character as to render medicine and advice unnecessary.

In the month of April I called upon a homœopathic physician of Philadelphia, as my breathing became affected and my voice very weak. Each respiration sounded as if coming from two individuals. He told me that I had an inflammation of the windpipe, and attended me until the eighth day of May.

On the day previous, I became so weak and exhausted from walking, or the least exertion, that confinement to my room became necessary, and my agony was so great that he was sent for in the night. His powders produced no effect. At 4 o'clock in the morning he pronounced me in no danger. I had spasmodic respirations which could be heard over the whole house. My eyes were fixed, and it was impossible to obtain sufficient breath to fill my lungs. I found that no position would give me any ease or comfort. It seemed as if every muscle of my face, chest and arms, was thrown into activity without giving me the least relief. I attempted to eat and drink, but found it impossible. The doctor gave me emetics and anodynes, but they produced no effect. He left me at six o'clock. At about 7 o'clock I became insensible.

Dr. Joseph Pancoast, of Philadelphia, reached me between the hours of 9 and 10 o'clock on the morning of the eighth May. He pronounced the disease "acute laryngitis." My pulse and respiration had ceased, and my face was black. He pronounced me dead! that it was too late for any operation to save me, but soon consented to try, and made the first incision without my showing any signs of animation. A discharge of very thick, black clotted blood very slowly followed. All the fresh air which was possible was admitted to the room. A slight twinge was observed of a muscle of the mouth, and then a slight heaving of the chest displayed a partial respiration, which was gradually increased until the whole mechanism of the body was again in motion. The blackness of the face gradually rose upward from the chin, like the removal of a black vail.

My feet were placed in warm water, a mustard poultice applied to the stomach, and brandy and water placed to the mouth (in a teaspoon) which I could easily swallow. I remained totally insensible for five or six hours after the operation, and then very gradually aroused, but was not aware of the operation until the next day.

I was surprised at the number of strange faces around me, and supposed, from appearances, that I had been very ill, but the enjoyment of breathing with perfect ease, after such intense suffering, was so great a luxury that it rendered me perfectly satisfied with my situation, with but little inclination to make inquiry. We had no further trouble until the next day (Sunday, the 9th,) in the evening, when the orifice became clogged with mucus, and the parts were highly inflamed. I was soon relieved by the forceps opening the orifice. In the absence of Dr. Pancoast, his friend, Dr. Mutter, arrived, who immediately ordered fifteen or twenty leeches applied to the throat, and a medicine called "neutral mixture," a tablespoonful to be taken every hour. My pulse had run up to 130, but by the next morning the fever had abated and I was doing well. The orifice was so constantly clogged with the mucus as to require instant watching, and I am greatly indebted to the young gentlemen in Dr. Pancoast's office for their disinterested kindness and attention day and night.

For the first three days I took no nourishment but mineral water. Milk punch was found to excite my pulse too much. I was then allowed arrowroot for several days, with lemonade and weak claret. In about three days after the operation, Dr. Pancoast applied the sponge attached to whalebone, soaked in nitrate of silver, ten grains to the ounce. It was very severe, on account of my weakness. This application was made once or twice a day, and afterward increased to twenty grains to the ounce.

On Friday, the 14th May, six days after the operation, I again began to suffer for want of breath, as the orifice had begun to heal so rapidly that the passage was nearly closed, and I had no power to take breath through the natural passage. Up to this period we had hopes that a cure might be effected without the tube. Dr. Pancoast reopened the orifice and inserted the tube, when I was instantly relieved. During the second week my throat was swabbed in the morning with nitrate of silver, and in the evening with glycerin. A tablespoonful of syrup sarsaparilla and hydriodate of potash was taken three times a day. This was continued until the fourth week, when a solution of lapis divinus was applied with the sponge and whalebone: the latter at noon, and the others as before.

After three weeks of confinement to my bed, I sat up a little at a time each day, and was able in the fourth week to ride and walk out. My appetite was very good, and from light diet I gradually advanced to the most hearty food, and have not eaten or drank anything which appeared to do me any harm. Near the expiration of the fifth week I was able to go to Providence, in company with Dr. W. D. Southall, of Richmond, Va., who has been constant in his attention to me, and to whom I feel under lasting obligations for his indefatigable kindness from the day of the operation until my case was resigned to your hands.

After my arrival in Providence, you continued for the first two weeks (which were the sixth and seventh weeks since the operation) with precisely the same treatment as that of Dr. Pancoast, in Philadelphia: in the morning a swab of nitrate of silver, twenty grains to the ounce; at noon a swab of the solution of lapis divinus; and at night a gargle of the glycerin. I also continued a tablespoonful of the syrup sarsaparilla and hydriodate of potash three times a day.

The seventh week, the strength of the nitrate of silver was increased to thirty grains to the ounce, and an alterative pill of biniodide of mercury.

The eighth week continued the same, with the exception that we tried the lapis divinus in the morning in lieu of the nitrate of silver, and found it more effectual.

The ninth week continued the lapis divinus in the morning, and at noon and night a gargle of cod-liver oil, continuing the pill every three or four nights, and taking half a tablespoonful of syrup of sarsaparilla and hydriodate of potash three times a day.

The tenth week continued the same.

I have taken great care not to expose myself to the weather. I have particularly avoided the night air, and in damp weather been carefully protected. Residing in the country I have found beneficial, but of all the remedies, I have found none that appeared to do me more good than breathing the pure *salt air.* My lungs are perfectly sound, and consequently did not find it too bracing, but each respiration seemed to invigorate and refresh me.

Experience has proved that a crowded room would do me great injury. On one occasion I entered a large church, which was about two thirds filled, and although sitting near an open window for about two hours, my exhaustion was so great on going out, that I was scarcely able to inhale a breath for several minutes. Walking faster than the ordinary gait, walking up a hill or up stairs, produces a shortness of breath and wheezing in the throat. I have an excellent appetite, and have no difficulty in eating or drinking, nor has anything taken into the stomach done me the least injury. I am disturbed but two or three times in the course of a night by a single cough, which discharges the mucus through the tube. I find it of the utmost importance that my mind should be kept perfectly calm and free from excitement. Smoking a cigar I find very soothing to the throat. There is a sufficient quantity of air passing up through the natural passage, to enable me to smoke as comfortably as ever.

I feel under great obligations to Dr. Pancoast for his promptness and skill in the operation which raised me from the dead, but nothing that I could say would add to his exalted reputation. His kindness will ever be remembered.

I will say a few words about the *tubes*, as my experience may be of benefit to others who are *blessed* in the same manner as myself. My first tube was inserted on the 14th day of May, about one week after the operation, and gave me immediate relief in breathing. It was only intended to be temporary, as there was no orifice in the back of it, to allow the passage of air up through the trachea. In the course of a week I had another tube made of the same diameter and length, with an orifice in the back, and by placing my finger over the mouth of the tube, and allowing my breath to pass through it and the orifice, I was enabled to speak so as to be understood by all around me. I soon found, however, that although the orifice was effectu[illegible]

in producing sound, it was so large as to take in the fleshy parts, which brought on bleeding and soreness, and in a few hours the tube would be so completely clogged as to require to be taken out. A third tube was made, but through mistake it was only three-fourths the size of the others, and I found it too small for breathing purposes.

I have tried various experiments with the size and position of the orifice in the back of the tube, and found that it would not bear to be more than two and a half lines long and one wide, and an oval shape was preferred; it should be cut about three-quarters of an inch from the plate. The fourth tube was made in this manner, and answered very well.

The opening in the throat has now so much healed, that I find it utterly impossible to breathe without the tube, and a duplicate of the fourth tube was made to be inserted as soon as the other was taken out. These I have been enabled to wear, only changing them night and morning. I place a piece of linen cloth, of the size of the plate, upon the back of it, covered with sweet oil or cod-liver oil, which prevents the plate from chafing the throat.

When the tube was first entered, I could only sleep well by lying upon my back. By turning upon either side, the clicking produced a hacking cough. This, however, was soon overcome by habit and experience, and I now find no difficulty in lying in any position.

A cough is often excited in changing the tubes, occasioned by the loosening of the mucus around them. The cough is immediately relieved on the discharge of the mucus. Some care is now necessary in inserting the tubes, in consequence of the healing of the orifice in the throat, it having diminished in size. I consequently prepare the duplicate tube, and have it ready to insert immediately after extracting the other, and before inhaling a breath, when the orifice is completely open. Having inserted the end of the tube, and waiting a few seconds for the spasmodic action to subside, it will pass home without any difficulty. If the orifice in the throat should be closed before the duplicate tube is inserted, I use the small silver forceps, which you designed for the purpose, and which I have constantly with me, as in case of accident they will afford relief until assistance can be obtained. These will easily enter the orifice and distend it, that the tube may enter, as well as if it had not been closed.

The tube may be easily cleaned by soaking it in water for a few minutes, when the mucus is softened, and a piece of whalebone and rag are passed through it. Great care should be taken that it be not bent or unsoldered; and I would here advise that all similar tubes be flanged upon the front side of the plate, and then passed through it when it may be soldered. My tubes are simply soldered upon the back of the plate.

On one occasion you will remember having been sent for, as the tube would not move all the way in, when you discovered that it had become unsoldered, and was merely hanging by a thread.

By placing my finger over the mouth of the tube, I am able to converse without pain or inconvenience, except that a strong effort is required, which would render a long conversation very fatiguing.

You have now a full history of my case and my experience, and I trust as my general health is now perfectly good, that with *your* usual kindness and attention, together with a due amount of patience and perseverance on *my own* part, we may yet be able to overcome the difficulty.

I remain, dear sir, yours very respectfully, W. E. H.

Providence, July 24, 1852.—*Boston Med. and Surg. Journal.*

